# Evidence-based orthopaedics: A brief history

**DOI:** 10.4103/0019-5413.40244

**Published:** 2008

**Authors:** Daniel J Hoppe, Mohit Bhandari

**Affiliations:** Division of Orthopaedic Surgery, McMaster University, Hamilton, Ontario, Canada

**Keywords:** EBM, evidence, evidence-based medicine, history

## Abstract

Evidence-based medicine was recently noted as one of the top 15 most important medical discoveries over the past 160 years. Since the term was coined in 1990, EBM has seen unparalleled adoption in medicine and surgery. We discuss the early origins of EBM and its dissemination in medicine, especially orthopaedic surgery.

## INTRODUCTION

The British Medical Journal compiled a list in 2007 of the 15 most important medical milestones since the journal's inception in 1840.^1^ Included were the discovery of DNA, the development of vaccinations and of antibiotics, the use of anesthetics for surgery and the emergence of evidence-based medicine. Evidence-based medicine (EBM) is an approach to the practice of medicine, whose name was coined by Gordan Guyatt in 1991^2^ and which was described by the Evidence-Based Medicine Working Group at McMaster University in 1992.^3^ It was a new paradigm that placed less emphasis on expert opinion and unsystematic clinical observations, instead stressing the impact of evidence derived from clinical research, such as randomized-controlled trials and the need for physicians to make themselves aware of published results before blindly accepting dogma.

Subsequently, there has been an explosion of research papers expanding the boundaries of EBM into many specialties of medicine, even including traditional Chinese medicine.^4^ The medical and health communities have embraced this methodology with great enthusiasm, to such an extent that one would be hard-pressed to find a physician today who has not heard of the term, EBM! In orthopaedics, the terminology collectively referred to as Evidence-Based Orthopaedics has also become a standard language of journals and major orthopaedic societies such as the Indian Journal of Orthopaedics, Journal of Bone and Joint Surgery, Clinical Orthopaedics and Related Research and Acta Orthopaedica. Furthermore, EBM has also evolved from an initial focus only on the best available published evidence for a treatment to the present emphasis on the importance of patient values and expected outcomes on management and treatment of disease.^5^

## WHAT IS EVIDENCE-BASED MEDICINE?

**Figure 1:**
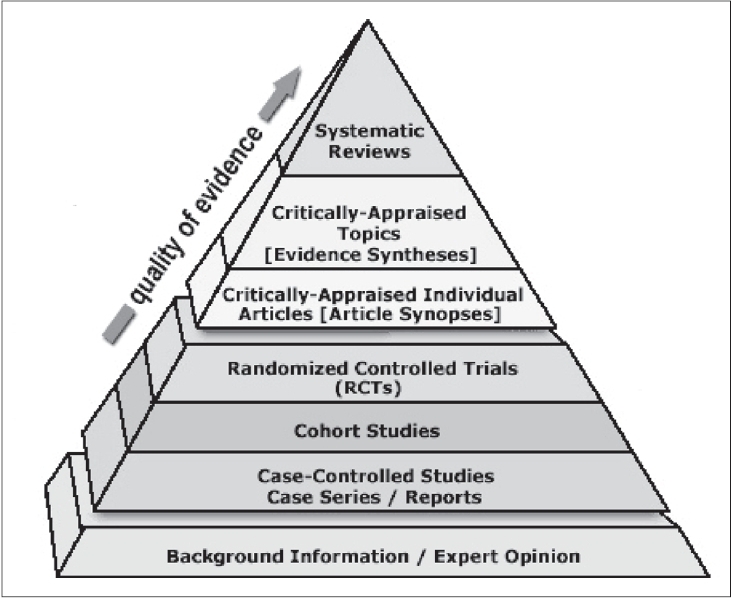
The EBM Pyramid of Evidence. The Sladen Library and Center for Health Information Resources. Downloaded from: http://sladen.hfhs.org/library/staff/ebm-resource-pyramid.htm

It is important to begin with an understanding of what we mean by Evidence-Based Medicine. Simply put, it is the integration of the best available research evidence, our clinical circumstances and patients' values and preferences. It can be described as a partnership between two components of the practice of medicine. One component represents the body of knowledge that includes all evidence, whether arrived at from physiological experimentation, individual observation and expert opinion, randomized controlled trials, systematic reviews or meta-analyses, as described by Sackett *et al*.,^5^ in 1996, can be visualized as a pyramid of evidence as shown in [Figure 1]. It is the objective and accumulated scientific and statistical wisdom derived over time that treats medicine as a scientific endeavour and demands that the user seek out the best available evidence that has been validated experimentally and statistically. It must also be recognized that some physicians may be talented diagnosticians with an acute intuitive ability to diagnose correctly. This ability may represent merely a high level of recall of scientific facts and the ability to connect knowledge to symptoms without the need of explicit logical steps.

The other component posits that such evidence alone is inadequate for making medical decisions for individual patients and that each patient's (and perhaps society's) values need to be taken into consideration and that the choice of treatment must involve both patient and physician. For instance, Guyatt^2^ contrasts two patients, both with pneumococcal pneunomia for which scientific evidence points to antibiotics as the best treatment, but where does the context of the patient comes to the forefront as a critical factor in the decision whether to treat with antibiotics or not? In addition to values, this side of EBM also relies on outcomes, namely weighing of treatments according to how they will translate into improved quality of life in a patient.

The synthesis of this partnership between brain and heart is EBM, which has matured in its fifteenth year into an established set of principles and guidelines for the practice of medicine, as found for instance in the Users' Guide to the Evidence Series in JAMA.^6^

## THE EARLY PRACTICE OF ORTHOPAEDICS: OPINION OVER EVIDENCE?

Consider the lead article in the Proceedings of the American Orthopaedic Association in 1889 with the title “Hypertrophy of One Lower Extremity”,^7^ in which the author, an orthopaedic surgeon, presented a case at a professional meeting that described treatment of his patient, a six year-old child, with a (diseased) leg three-quarters of an inch longer than the other. He prescribed application of a rubber bandage but the diseased leg's growth continued to outpace the other and after a year he recommended only that the child wear a high shoe on the better leg for comfort. The surgeon noted small cysts deep beneath the skin but did not observe any inflammation of the skin that might be connected to elephantiasis. The patient was later examined by another specialist who diagnosed congenital occlusion and dilation of the lymph channels and recommended amputation, which was carried out. Following post-surgery tissue examination the author thought that the limb growth could be accounted for by retention of lymph caused by parasite, in the same way that the presence of parasite filaria and eggs cause obstruction in elephantasis.

Essentially, this author has presented an unsystematic clinical observation on the subject,^8^ which he has shared with other physicians. He has also attempted to find a cause for his observations through a pathological investigation of post-mortem tissues where he has been influenced by knowledge of a different condition that manifests similar symptoms.

His presentation, written up for journal publication, was followed with a discussion by other specialists. One described a patient, a twenty-one year-old female with a similar increase in size in one leg for whom he also prescribed a high shoe. He “did not know what diagnosis to make” and referred the patient to another surgeon who also confessed “ignorance of the nature of the problem.” A second discussant mentioned a similar case that he treated by stretching the sciatic nerve, which reduced the size of the affected limb. His choice of treatment was motivated by a case of elephantiasis a decade previously in which he removed an inch of the sciatic nerve with a subsequent lessening of the size of the patient's calf.

These surgeons, all from different cities, offered radically divergent treatments for a particular type of affliction: shoe lifts, sciatic nerve stretching and amputation. As this journal was the official organ of the American Orthopaedic Association, one would expect it to provide the best source of information to practitioners. But how could a reader decide which treatment to adopt if he were to encounter a similar case in his own practice? Which expert was he to believe? And what if he didn't subscribe to this journal or attend the meeting? How could the information presented be transmitted?

It is clear that each of the discussants offered their expert opinions. That is why they were discussants. Their knowledge was based on observations in their own practices on a case-by-case basis, which reinforced their understanding of the value of a technique or treatment. Such knowledge would then be transmitted orally to students through mentoring or to other doctors in clinical rounds or in discussions following pathological examinations. The wisdom that is imparted this way sometimes results in aphorisms such as “If you hear hoofbeats, think horses, not zebras” although, as pointed out by Groopman in his recent best-seller “How Doctors Think”,^9^ when a physician's reasoning is unduly influenced by what is thought to be typically true, regardless of the evidence, errors in diagnosis may sometimes result. When experts differ, a physician may be swayed by the opinion of the physician with whom one is better acquainted or by whoever has the stronger reputation or comes from the more prestigious institution.

Interestingly, one contributor to the discussion recognized the need to reconcile the different opinions and raised the following suggestion: “Would it not be in accordance with the purposes of this Association to appoint a committee to investigate this subject, taking patients… and treating them.” The implication is that anecdotal evidence based on individual cases or experience is insufficient evidence to adjudicate the efficacy of a treatment and instead trials are needed to objectively demonstrate the benefit of one cure over another. Could it be that a century ago the importance of large clinical trials was being recognized?

In addition to expert opinion, a physician, searching for understanding, would also be influenced by his acquaintance with experimental physiology. Knowledge of how the body functions and reacts to stimuli or foreign matter is derived from laboratory experiments, generally on animals, to validate or invalidate hypotheses. This scientific method is the basis for research in the biological, physical and chemical sciences. The discovery of penicillin by Alexander Fleming in 1929^10^ is an illustration of how an accidental observation of differential bacterial growth in a Petri dish, stimulated by scientific curiosity, led first to the development of penicillin and then to an understanding of the physiological basis of antibiotics. In this first article of the journal cited above, the author did, in fact, also attempt some experimentation by carrying out post-mortem tissue examination in order to find clues to the cause of his patient's condition and thereby increase his understanding of the problem for future cases.

## LAYING THE FOUNDATION FOR EBM: EARLY CLINICAL TRIALS

The manner in which medical knowledge develops has changed over time. As Geoff Watts has written: “Knowledge doesn't suddenly appear in neat and tidy quanta. Like patches of lichen spreading over a rock face, it accretes over decades”.^1^ Each key development is built upon by earlier ideas.

Clinical trials are not a recent research tool. There is actually evidence of what may be the first clinical trial in the biblical book of Daniel, describing events that occurred over 2600 years ago. Neuhauser and Diaz^11^ provide a refreshing look at the “original clinical trial” in their article on the subject in 2004. Basically, King Nebuchadnezzar wanted the Israelite children to eat a diet of the king's meats and wines. The prophet Daniel, believing (hypothesizing) that a diet of beans, lentils and water would be healthier than the king's diet, formed an experimental group of himself and three other children and asked to be compared to the rest of the children after a 10-day trial of his diet. Indeed, after 10 days had passed, the experimental group was compared with the other children (the “control group”) and “their countenances appeared fairer and fatter in flesh than all the children which did eat the portion of the king's meat”.

Two famous clinical trials were carried out in the 18^th^ century, trials on scurvy and on smallpox. Many physicians are aware of the work by Sir James Lind in the prevention of scurvy in 1747.^12^ Lind divided 12 similar patients with scurvy into six groups of two each and placed each group on a different diet. One group received oranges and lemons. In effect, Lind was carrying out a one-way analysis of variance. He found that oranges and lemons provided the best treatment.

The discovery by William Jenner of using cowpox vaccine to immunize against smallpox was preceded by both clinical observations and clinical trials. Inoculations had been around for centuries, dating back to 10^th^ century China and India,^13^ but their use had been justified based on clinical observation that those inoculated were less sick than the infected. In China, these were performed by placing cotton soaked in infected pus into subjects' noses.^14^ Cotton Mather, a colonial minister living in Boston during the smallpox epidemics of the 1720's, had seen such practice firsthand during time spent in West Africa. He convinced a local physician to inoculate his patients, although many people in America were against it, including all of the local physicians. In fact, the townspeople were so incensed that a bomb was thrown into Mather's house, even though it did not end up exploding. However, Mather persevered and he tallied and compared the mortality rate of those who were inoculated with the local population. In total, 6 out of 287 (2.1%) inoculated patients died compared with 842 deaths out of the 4917 (17.1%) who received no treatment.^14^ This type of study would be what is now referred to as cohort trial, in which exposed and non-exposed groups of patients are followed forward in time and monitored for the occurrence of a predicted outcome, in this case mortality.^8^

Another step forward on the path to EBM was the introduction of the randomized-controlled trial into the medical literature (RCT). One of the first truly randomized trials in medicine was published in 1931 in the American Review of Tuberculosis by J Burns Amberson, a staff physician at Detroit Municipal Tuberculosis Sanatorium in Detroit.^15^ He divided 24 patients into two groups of 12, based on approximately matched pairs. By flip of a coin, one group became control, treated with injections of distilled water and the other was treated with sanocrysin, a gold preparation. This is an example of a randomized block design and although the results showed no therapeutic benefit,^16^ the methodology was important for setting a standard in how clinical trials should be undertaken.

Two decades later, another randomized controlled trial was carried out by the Streptomycin in Tuberculosis Trials Committees of the British Medical Research Council.^17^ This was a multi-centre, double-blinded clinical trial and served as a model for future designs. The patients were randomized by using numbered envelopes and their progress was evaluated through monthly chest x-rays read by three specialists who did not know whether the patients had received streptomycin or the control, which was bed rest. The results of this clinical trial showed that the death rate was significantly lower for patients receiving streptomycin.

Perhaps the largest single clinical trial carried out in the 20^th^ century was the Salk polio vaccine field trial in 1954.^18^ It was understood at the time how the poliomyelitis virus entered the system and how it affects the central nervous system causing paralysis and sometimes death. It was also observed that severe polio was rarer in communities with poor hygiene, leading to the hypothesis that children in these communities were conferred immunity by mild exposures to the virus. Because polio was so rare, an enormous number of participants (over 400,000 children) were needed for the trials in order to observe any possible significant effect. Roughly half were vaccinated and half received a placebo of salt water. The results confirmed the effectiveness of the Salk vaccine and led to large-scale inoculation of school children. This RCT was also double blind so the examining doctors would not bias their diagnoses. Such a trial is considered the gold standard of designs.

As randomized controlled trials became more common, studies were published that contradicted conventional wisdom, thus showing the necessity of making clinical decisions based on evidence rather than on observation and physiological principles. An outstanding example of this is the discontinuation of hormone replacement therapy (HRT) after the 2002 Women's Health Initiative (WHI) trial.^19^ HRT had been recommended since 1985 for prevention of osteoporosis, dementia and heart disease, as well as to improve the general quality of life of postmenopausal women. The basis of this recommendation was clinical observations that women taking HRT seemed to be healthier than those not taking it. Pharmaceutical advertising influenced physicians in prescribing it for their patients and there were some attempts to explain the benefits physiologically using laboratory experiments. Unfortunately, the WHI trials showed increased risk of heart disease, breast cancer and stroke in women taking HRT and the trials were actually stopped early. The WHI trials took place about 10 years after the introduction of EBM and are a striking example of the effect of EBM on medical thinking.

However, most clinical trials were not of the magnitude of these large trials and involved few patients, which sometimes produced conflicting or inconclusive results. Clearly, something needed to be done to assess the quality of each study (now called critical appraisal) and a quantitative method needed to be developed to draw conclusions from the results of multiple studies on a single topic (statistical reviews and meta-analyses).

## CRITICAL APPRAISAL: THE LATE 1970S AND EARLY 1980S

The ingredients of EBM that emphasize finding the “best” evidence from the literature were already taking root and practiced at McMaster University in the late 1970s and early 1980s by David Sackett, who used the term “critical appraisal” to describe the systematic examination of the medical literature to extract evidence. The term “Evidence-Based Medicine”, however, was actually coined by Professor Gordon Guyatt in 1990 in a brochure for internal medicine residency applicants to McMaster University. In this early description, EBM was described as an “enlightened skepticism” towards the use of diagnostic, prognostic and therapeutic technologies. The result of this early work was a series of “Readers' Guides” articles by McMaster colleagues in the Canadian Medical Association Journal^20^ followed by several texts.^21^,^22^

The initial intention of EBM was educational, to train residents to become better physicians. This was consistent with the philosophy underlying the unique approach to medical education at McMaster's nascent M.D. program and the university's focus on innovation in education, an emphasis that remains today. It also recognized that physicians in a busy practice have limited leisure time to peruse the literature and part of the training was concerned with efficient methods for extracting information from literature in a timely fashion. Soon, faculty became intrigued by what their students were learning and also became interested in understanding this new approach. The advent of microcomputers around this time also gave an impetus to facilitating searching, although not to the incredible extent that physicians are able to locate information today through the internet and associated electronic searching capabilities of databases, document repositories and documents themselves. As well research can now be published online immediately avoiding the lag in the past between completion of a paper and its distribution.

Membership in the group of physicians interested in critical appraisal increased to encompass physicians in Clinical Epidemiology and Biostatistics, Medicine, Obstetrics and Gynecology, Pediatrics and Emergency Medicine, not only at McMaster, but elsewhere. This group evolved into the Evidence-Based Working Group and culminated in adoption of the term EBM and publication of the fundamental paper^3^ announcing this approach as a new paradigm.

## ADVANCES IN META-ANALYSIS AND SYSTEMATIC REVIEWS: THE MID-1980S

During the period that the McMaster group was emphasizing the importance of examining the literature in understanding the efficacy of medical treatments, a similar revolution was occurring in the social sciences^23^ with the development of meta-analysis. It originated as quantitative research tool to allow researchers to combine and synthesize the results of a large number of separate studies in order to gather evidence pertinent to a particular topic in the hope that, taken together, the data as a whole would either confirm or dispel a claim (or hypothesis).

Meta-analyses gain power by pooling the results of many small trials and help determine whether a suggested treatment shows clear evidence of effectiveness; whether the results, though inconclusive, merit additional trials because the treatment appeared promising or whether it should be altogether abandoned.

The importance of meta-analysis in medicine was clearly identified in a seminal book by Chalmers, Enkin and Kierse.^24^ These authors searched the literature to gather information on an enormous number of randomized clinical trials and then organized teams of physicians to assess the research quality of the trials and to carry out meta-analyses on various suggested treatments. Recommendations for physicians and nurses were described in a less technical publication.^25^ Meta-analyses, are now increasingly carried out in medicine, for instance in orthopaedics^26^ and provide one of the highest levels in the hierarchy of evidence.

## COCHRANE COLLABORATION: THE 1990S

Although out of the scope of pre-EBM developments, no discussion of EBM would be complete without mention of the Cochrane Collaboration, a leading source of reviews. The first Cochrane Centre was established by Ian Chambers at Oxford University in 1992 in response to Archie Cochrane's rebuke of the medical profession for not having established a database, to be regularly updated, of published clinical trials according to specialty. This was followed by the second Centre at McMaster University in 1994, leading to the Cochrane Collaboration, which has been a repository since 1996 of systematic reviews and critical appraisals of the medical and health literature as part of the Cochrane Library. Jadad *et al.*, published a comparison of Cochrane reviews with published articles, which showed that Cochrane reviews are more rigorous methodologically and are more frequently updated than systematic reviews or meta-analyses published in journals.^27^

## THE EVOLUTION OF EBM IN ORTHOPAEDICS: THE 21^st^ CENTURY

We began this paper with a discussion of the first article in the Proceedings of the American Orthopaedic Association. Fast forward from this initial illustration of 19^th^ century medical learning to the year 2000, a full century later. This same journal, now named the Journal of Bone and Joint Surgery, in recognition of the need to integrate clinical expertise with the best available systematic research, introduced a new section, “Evidence-Based Orthopaedics”.^28^ In the introduction to this new section, the editors wrote that randomized clinical trials would form the main contribution because they are believed to provide the highest quality evidence and therefore when available should influence clinical decision-making. Many of the articles now published in this section are systematic reviews and meta-analyses.

Among the early papers to appear in this section were a series of four User's Guides to the Orthopaedic Literature^29^–^32^ by Bhandari, Guyatt and their collaborators, covering prognosis, surgical therapies, diagnostic tests and literature reviews. These articles were meant to teach orthopaedic surgeons the manner in which evidence can be evaluated and applied in their practice. Each of these guides begins with a scenario describing a patient of an orthopaedic surgeon with a particular condition, ending with the patient asking question to which the doctor does not know the answer or the doctor weighing two or more possible treatments.

For example, in the first User's Guide,^29^ the scenario involves a patient with a displaced distal radial fracture. Based on the patient's age and fracture type, the surgeon decides that it warrants a closed reduction. However, a colleague points out that such fractures are prone to instability and suggests using a new type of bone cement. The surgeon decides to search the literature during the five hours before the OR is free in order to formulate a decision. This article then details how the surgeon would perform a literature search and the steps needed to critically appraise the articles found based on the quality of the design, whether the results are valid and whether the results are applicable to the patient at hand. Many of the points made are not obvious and the authors provide a detailed prescription that can be used not merely for the case scenario presented, but as a template for surgeons in general trying to understand how to use an article about a surgical therapy.

In 2003, the Journal of Bone and Joint Surgery decided that all clinical articles submitted for publication would have to include a level of evidence rating to classify the quality of study.^33^ They chose five levels, the lowest being expert opinion and the highest being RCTs or systematic reviews of RCTs and separated the articles into four types, in order to make it clear to the reader what the purpose of an article was. The importance of these measures was two-fold: to facilitate review by the editors and to enable surgeons to more readily assess the quality of evidence and what weight to give to a study before incorporating the results into their clinical practice.

Yet, despite these refinements in the presentation of knowledge, many surgeons do not have the time to sift through even well-formulated articles and it is not clear that they would be able to translate current research into better care for their patients.^34^ As a result, there is a great need for systematic reviews and meta-analyses. Similar to the User's Guides, Acta Orthopaedica has launched a series of articles on evaluating meta-analysis^35^ and critical appraisal^36^ to assist surgeons in this respect.

Finally, in a very recent article published February 2008, the Osteoarthritis Research International (OARSI) group published the second of two articles^37^,^38^ describing their recommendations for the management of hip and knee osteoarthritis, which they arrived at through a critical appraisal of existing guidelines, a systematic review of the recent research evidence and the consensus of a body of multi-disciplinary experts in primary care, rheumatology orthopaedics and EBM. It was interesting that EBM itself was considered its own specialty, together with the other three traditionally accepted specialties. This particular study represents the highest level of evidence that is possible from the current state of knowledge. It combines expert opinion with clinical trials and systematic overviews as part of a large team effort.
